# Perspectives of patients with type 1 or insulin-treated type 2 diabetes on self-monitoring of blood glucose: a qualitative study

**DOI:** 10.1186/1471-2458-12-167

**Published:** 2012-03-08

**Authors:** Johanna Hortensius, Marijke C Kars, Willem S Wierenga, Nanne Kleefstra, Henk JG Bilo, Jaap J van der Bijl

**Affiliations:** 1 Diabetes Centre, Isala Clinics, Dokter Spanjaardweg 11, PO Box 10400, 8000, GK Zwolle, the Netherlands; 2 Nursing Science, University Medical Center, Heidelberglaan 100, Utrecht, the Netherlands; 3 Clinical Psychology, Meander Medical Center, Ringweg Randenbroek 110, Amersfoort, the Netherlands; 4 Department of Internal Medicine, University Medical Center, Hanzeplein 1, Groningen, the Netherlands; 5 Medical Research Group, Langerhans, p/a Dokter Spanjaardweg 11, Zwolle, the Netherlands; 6 Faculty of Health, Welfare and Sports, Inholland University of Applied Sciences, Boelelaan 1109, Amsterdam, the Netherlands

## Abstract

**Background:**

Self-monitoring of blood glucose (SMBG), including self-regulation, is an important tool to achieve good glycemic control. However, many patients measure their glucose concentrations less often than is recommended. This study investigates patients' perspectives of SMBG and all relevant aspects influencing SMBG in patients with type 1 and insulin-treated type 2 diabetes.

**Methods:**

In depth interviews were conducted with 13 patients with type 1 diabetes from an outpatient clinic and 15 patients with type 2 diabetes from general practices. All interviews were transcribed verbatim and analyzed using the Grounded Theory approach.

**Results:**

A wide variety of SMBG was encountered. Perceptions, goals of SMBG and personal and contextual factors were identified, influencing the respondents' perspective of SMBG, and leading to this variety. Respondents experienced a discrepancy between their own and the professionals' perceptions and goals. Respondents' perception of SMBG ranged along a continuum from 'friend' to 'foe'. With respect to the goals, the respondents experienced tension between achieving good glycemic control and quality of life, and deliberately made their own choices. The performance of SMBG was tailored to their perceptions and personal goals. Personal and contextual factors such as hypo- or hyper (un)awareness, knowledge, and contact with professionals acted as either facilitating factors or as barriers to SMBG, depending on the respondents' perspective. A SMBG model was developed providing a representation of the factors and their interrelations.

Respondents with type 1 diabetes seemed more resigned to their situation and SMBG was more integrated into their lives.

**Conclusions:**

From the patients' perspective, professionals positively present SMBG as a 'friend' in order to achieve strict glycemic control. Whereas patients can also perceive SMBG as a 'foe'. They primarily seek a personal balance between achieving glycemic control and quality of life, leading them to deliberately make other choices regarding SMBG performance than was recommended. Gaining insight and discussing all factors affecting SMBG will help professionals and patients come to mutually agreed goals and to tailor the performance of SMBG to the individual patient. This should result in a more optimal use of SMBG, an improved quality of life, and improved clinical parameters.

## Background

The Diabetes Control and Complication Trial [1] and the United Kingdom Prospective Diabetes Study [2] demonstrated that strict glycemic control significantly decreases the risk of long-term diabetes complications. In order to obtain good glycemic control, self-monitoring of blood glucose (SMBG) is essential in insulin-treated patients with diabetes [3-5]. SMBG includes an assessment of the capillary glucose concentration (self-measurement) as well as the interpretation of and responding to the readings (self-regulation). The goal of SMBG is to achieve blood glucose levels as near to normal as possible in order to prevent long-term complications, to be able to take adequate decisions in relation to diet, exercise, and medication, to evaluate the effects of these decisions, and to detect hypo- and hyperglycemia [6,7].

Many patients, however, monitor their blood glucose less than is recommended by their healthcare provider [8-10]. Quantitative research shows several barriers to SMBG. These include a longer duration of the disease, pain, low self-efficacy, low self-esteem, increased anxiety and depression, alcohol abuse, smoking, complex treatment regimes, decreased social supports, poor communication between patients and health care providers, lack of education, and lack of health insurance [8,10-16]. Qualitative research on patients' perspectives on SMBG, including the barriers and facilitating factors is scarce[17-20]. In these studies other barriers to SMBG have also been identified such as an increased awareness of their diabetes, physical discomfort, not understanding the relationship between SMBG values and the behavior of the patient, not knowing how to correctly respond to the glucose readings, and being in poor glycemic control. However, in most of these studies the focus is mainly on patients with type 2 diabetes, not treated with insulin.

More qualitative research is needed to increase the insight into perspectives of SMBG in insulin-treated patients with diabetes. These insights can help professionals support the patients in their self-management regarding SMBG [16,20,21].

The objective of this qualitative study is to investigate the perspectives of patients with type 1 and insulin-treated type 2 diabetes regarding SMBG, including the barriers and facilitating factors in performing SMBG, and whether differences in perspectives exist between patients with type 1 and type 2 diabetes.

## Methods

Our intent was to move beyond description and to identify and explain factors which affected the patients' perspectives of SMBG. The grounded theory approach provides a methodological framework to develop theory, including a model, that interprets the data [22-24].

### Patients

Patients with type 1 diabetes were recruited from the outpatient clinic of a general hospital in the Netherlands, and patients with type 2 diabetes from general practices in the same region (Isala Clinics Zwolle, the Netherlands). Eligibility criteria were: a diagnosis of type 1 or type 2 diabetes, treated with insulin, SMBG carried out for a minimum of one year, Dutch speaking, and over the age of 18.

The initial selection focused on building a patient population with as much variation as possible in the factors relating to SMBG. Physicians and diabetes specialized nurses were asked to select patients who differed in age, gender, living status, education, type of diabetes, insulin therapy, duration of SMBG, and employment. When the patient expressed an interest in participation, printed information was provided. The researcher then followed up by telephone to answer any remaining questions and to make an appointment with the patient. The patient population ultimately consisted of 28 patients, 13 patients with type 1 and 15 patients with type 2 diabetes. The demographic and background characteristics of the patients are presented in Table 1.

**Table 1:** Characteristics of the patients

Description	DM 1 (n = 13)	DM 2 (n = 15)
Male	5	8

Age (years)	45 (40-58)	71 (60-76)

Marital status		
Married/cohabiting	11	11
Single	2	4

Education:		
Low^a^	3	10
Middle^b^	8	5
High^c^	2	

HbA1c (mmol/mol)	57 (55-63)	58 (53-66)

Diabetes duration (years)	19 (13-28)	11 (5-16)

Type of insulin therapy:		
1 insulin injection per day		7
2 insulin injections per day		3
4 insulin injections per day	3	5
insulin pump	10	

Duration of SMBG (years)	19 (13-24)	8 (4-13)

Data are n or, due to skewed distribution, median (P25, P75)

^a^Low: primary school, lower secondary general, lower vocational

^b^Middle: higher secondary general education, intermediate vocational

^c^High: higher vocational education, university

The patients were informed that their anonymity was guaranteed and that all information gathered would be confidential by disassociating the patient name from the data. Each patient received a research number, and the data were saved under this number. Approval for the study was obtained from the Medical Research Ethics Committee of the Isala Clinics in Zwolle (NL 27433.075.09). All patients provided written informed consent.

### Data-collection

Data were collected through the conduction of one-time open in depth interviews, guided by a topic list. This topic list was compiled based on literature, including the General Self-management Model which is based on the Chronic Care Model [25,26] as well as topics gathered during two focus group meetings. These meetings were held with patients who attended the same outpatient diabetes clinic in a hospital setting. The first group consisted of eight patients with type 1 diabetes: five men and three women with a median age of 52 years (Interquartile range (IR):37-61), and a median diabetes duration of 25 years (IR:5-39). The second group consisted of eight patients with type 2 diabetes: four men and four women with a median age of 68 years (IR:48-76), and a median diabetes duration of 10 years (IR:9-16). The focus group interviews were led by the principal researcher (JH) and a psychologist (WSW). These interviews also increased the principal researcher's preparedness to conduct the individual interviews. Focus group patients were not included in the individual interviews.

The following topics were incorporated in the individual interviews: the frequency of SMBG, its goals, the effect on daily life, knowledge, patient skill level, confidence in self-care, living with diabetes, the patient's contribution to treatment, the role of relatives, the role of healthcare providers, as well as any barriers and facilitators for performing SMBG.

The question posed to the patients to initiate the in depth, open interviews was: 'What does it mean to you to perform SMBG?' A non-judgmental atmosphere was strived for with a clear emphasis placed on the fact that the investigator was there to learn from the patients. As the study progressed, the focus of the interviews shifted toward the specific issues which were identified throughout the data analysis. Theoretical sampling continued until no new ideas arose which were of value to the developing theory, and saturation was reached.

The interviews were held either at the patient's home, at the Isala Clinics or at the general practice according to the patient's preference. They were conducted by the principal researcher, an experienced nurse specialized in diabetes (JH). She had had no prior professional contact with the patients.

### Data-analysis

Interviews were tape-recorded and transcribed verbatim. MAXqda 2007 was used for the analysis of the interview data. The data-analysis consisted of three phases of coding [22-24]: initial coding by breaking down the data (open coding), reconnecting the broken data into categories (axial coding), and interconnecting the categories into a model that represents the factors related to the patients' perspectives of SMBG (selective coding). The coding and categorising involved constant comparison. The principal researcher coded and categorised the data. Three other researchers individually read the interviews and the coding results, and/or were involved in developing the theory. The researchers had different backgrounds and therefore contributed to alternative perspectives, thereby preventing going native. During meetings, consensus about the coding, categories and the developing theory was achieved. This researcher triangulation procedure served to increase the depth of the analysis and established increased validity. Memos were used in which the ideas were written down about the evolving theory. Validation was enhanced by member check and peer review. Four of the respondents were asked to verify the written summary of their interviews. All four confirmed that the summaries were a fair representation of their perspectives. Provisional conclusions and theoretical insights were discussed with a person from the Dutch Diabetes Association and a diabetes specialized nurse.

## Results

**Figure 1:**
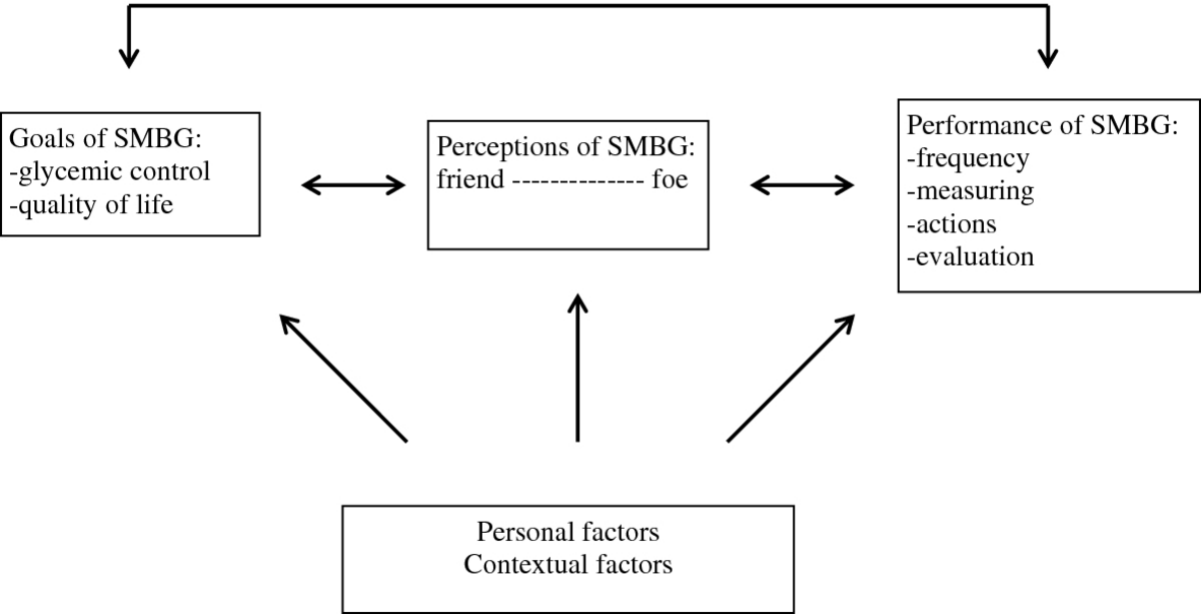
**Model: Self-monitoring of blood glucose**.

The respondents' perceptions, their goals, and several personal and contextual factors were identified as important factors which affected the patients' perspectives of SMBG. These factors led to a wide variety of performances, and did prove to be interrelated. In Figure 1, a SMBG model is presented which represents the respondents' perspectives, the influencing factors and how they interrelate. Our analysis revealed that the difference in perspectives between men and women was that feelings of shame towards health care providers about 'poor' readings were only reported by women. First, a description of SMBG in daily practice is provided followed by a review of the factors.

### Variety in SMBG performance in daily practice

The individual performance of SMBG differed with respect to frequency, timing, the actual measurement, interpretation of the readings, the resulting action taken, and an evaluation of the outcome. The respondents made their own choices regarding the performance, and they had their own reasons and logic for the choices they made. A wide variety in SMBG performances was seen in this study as a result. 15 respondents reported that their frequency of SMBG use was according to the recommendations provided by their health care provider, 3 respondents reported a lower frequency, 3 respondents reported a higher frequency, and 7 respondents reported that they had received no specific recommendations. In addition, a majority of all the respondents reported that their frequency and timing of SMBG varied from day to day.

'I measure my blood sugar every morning. If it's a bit low, around 6 or so, and I know that I don't have a lot planned for the day, then I don't worry about it. If we are going out somewhere, however, then I think about what we are going to do, and I measure it more often, and I will already have an extra slice of bread in the morning. I think ahead. I don't like feeling hypoglycemic. I want to prevent that. Even, if I am just going somewhere by car, I will do an extra measurement. Imagine if I was to get an hypoglycemia. There are already enough people in the ditch.'

Respondents differed in their interpretation of the readings, the subsequent actions taken, and their evaluation of the outcomes of these actions. For example, a high glucose concentration could lead the patient to adjust the insulin dosage, change the food intake, contact the health care provider or to take no action and adopt a wait-and-see policy.

'I had the self monitoring glucose meter, but I didn't do a lot with it. I figured that things were actually going pretty well. I measured my blood sugar about once per month, and if the results were good, then I thought: things are fine. If the results were not good, then I thought: well, what can I do about it? I don't know. I was being followed by both my family doctor and a nurse specialized in diabetes. My weight was good, I didn't have too much belly fat,... so I figured that things were going all right. So I just waited to see what the next month's results would bring.'

### The perception of SMBG: a continuum between 'friend' and 'foe'

Some respondents perceived SMBG as a 'friend', giving them confidence, freedom, certainty and peace of mind. It helped them to achieve their personal goals including good glycemic control, autonomy, control of their diabetes, and the ability to lead a normal life.

'I measure my blood sugar to see if it stays somewhere between 5 and 8. And it is the expectation that you check your own levels once a week. That is the agreement. That gives me a good feeling. Some people get very anxious when they have to prick their finger. Not so for me. On the contrary, it gives me a feeling of security.'

In respondents who were no longer familiar with the symptoms of hypo- and hyperglycemia, SMBG was felt to be helpful in prevention and detection. Most respondents, who perceived SMBG in a positive way, reported that it had become a habitual part of their daily lives.

Other respondents perceived SMBG more as a 'foe'. They mentioned many experiences to support this perception. For example, the finger prick can be painful and can lead to callous and hard spots.

'It is not easy to get a drop of blood. I have got hard black marks on his hand. The marks are so conspicuous. I sometimes find that I am confronted by them. When I am 60 or 70 years old I worry that I won't be able to get through the skin at all! And the idea that your fingers will be totally covered with callous...'

Some respondents felt obliged to monitor their glucose levels with SMBG. When they decreased the frequency of SMBG, their glucose regulation worsened, and they had a higher risk of developing an unnoticed hypo- or hyperglycaemia. Furthermore, when the readings were not in accordance with the expectations, especially unexpected 'poor' readings, respondents reported feeling frustrated, anxious, ashamed, or helpless. Some respondents felt that they were never free from their diabetes, always feeling that they had to focus on it. According to these respondents SMBG required a lot of organizational effort and interruption of their regular activities. They felt they had to carry a lot of things with them whenever they would go out in order to be able to measure their glucose concentration.

'Always having to think: did I remember this? Did I remember that? My pockets are always stuffed full of all kinds of things. It's annoying. My purse is like a moving van. I can't ever go out with a small neat purse. Not even when I go to the opera house.'

The respondents' perception of SMBG appeared to be a dynamic factor. For example, adjustments in materials e.g. another kind of finger-pricking device, could make blood glucose testing easier and less painful. This led to a more positive perception. The perception of SMBG as a friend or a foe was related, to some extent, to the frequency of measurement. Respondents who perceived SMBG as a foe, did not always measure their blood glucose concentration less often than those who perceived SMBG as a friend, but they did encounter more difficulties with the measurements.

### Personal goals: tension between good glycemic control and quality of life

From the interviews, it became clear that the respondents were not only focused on good glycemic control. They were also focused on maintaining their quality of life.

To achieve the desired glycemic control in order to prevent complications, respondents reported that they had to measure their glucose concentrations frequently in order to make the required adjustments in insulin dose, food intake, and lifestyle. Furthermore, these frequent measurements were necessary for the prevention and detection of hypoglycemia.

'My sugar levels are regularly on the low side. If you are tightly controlled, that's what happens. I am hypoglycemic at least once per day. I would rather live this way with a lower average than be hypoglycemic less often with a higher average. I always think that if your levels are high then the disease keeps nibbling pieces off of your blood vessels. It's like a time bomb which you can't give too many chances. The price is that you are then hypoglycemic with some regularity.'

Respondents described their quality of life in terms of maintaining autonomy, living a normal life, enjoying life, not having to always focus on their diabetes, not being considered a patient, and not wanting to be a burden to their relatives. The influence of quality of life on the frequency of SMBG was less clear than the influence of good glycemic control. It depended on how the respondents individually interpreted quality of life. For example, the concept of living a normal life could mean that the respondent did not want to focus exclusively on the diabetes, which would lead to measuring the blood glucose concentrations less often. However, it could also mean that the respondent would measure the blood glucose more frequently in order to keep the diabetes under control so that he/she would be able to do the activities he/she was used to doing.

Many respondents set their own personal target values with the goal of preventing either a hypoglycemia or a hyperglycemia. Many respondents experienced a hypoglycemic state as being quite distressing. They felt that it kept them from living a normal life and from having their diabetes under control. These respondents were willing to accept higher glucose readings. Other respondents primarily wished to prevent a hyperglycemia to prevent complications. They accepted being hypoglycemic more often.

'When I am hypoglycemic, I feel wretched. I even notice that I can become quite aggressive. I don't really have a problem with high sugar levels, but the low ones are quite bothersome. I really try to avoid attacks of hypoglycemia. I become quite anxious about them. You also don't want to get aggressive. It's very unpleasant and actually you totally don't want that.'

Achieving a good quality of life and satisfactory glycemic control were sometimes in alignment with each other. For example, respondents reported feeling better when their glucose levels were better. However, a majority of the respondents experienced tension between achieving both these goals simultaneously. They tended to deliberately make their own choices regarding the goals of SMBG, and tailored their performance of SMBG to these goals. They were aware of the discrepancies between their decisions and the recommendations they had received from their health care providers.

### Personal and contextual factors: barriers or facilitators

Personal and contextual factors may act as barriers or facilitators in the performance of SMBG, depending on the respondents' perspective. Furthermore, these factors could result in goal adjustments and a shift in the perception of SMBG rendering it more positive or negative for the patient.

#### Personal factors

##### Personality

Respondents explained that their performance of SMBG was a result of their personality make-up including such things as perfectionism, being down to earth, or being easily worried. Some respondents reported that these traits were influenced by their upbringing, such as not being allowed to complain. Their personality influenced their goals, their perceptions and their SMBG performance.

'I'm a bit of a perfectionist, and I aim for really good glucose levels. And then I'm really scared that if, one time, I do forget, that my sugar level will shoot up. So then you start self-monitoring again...'

##### Acceptance

Some respondents mentioned that they had trouble performing SMBG as it forced them to confront and accept their diabetes in a concrete and visible way. One respondent reported that psychological factors played a role, as he did not want to have to make changes in his life or give things up. As a result, he measured his blood glucose less frequently.

##### Depression

Some respondents reported having been depressed. This did not always directly influence their SMBG behaviour, but it did influence the goals surrounding their measurements. They did tend to accept higher glucose concentrations. They strived for keeping control of their diabetes by preventing hypo- and hyperglycemic symptoms. They were not able to strive for optimal glycemic control.

'In that period of depression I was just happy when I felt good and that things were moving again, and that I could do my job again and things like that, and for me that was enough. The diabetes just wasn't that important for me. I actually made the choice to just let it be there for what it was. Not that I became negligent about it, absolutely not, but... well, slowly but surely, as my life got back on track and other things became more normal again, then I could start refocusing on the diabetes.'

##### Awareness of hypo- and hyperglycemic symptoms

The loss of awareness of hypo and hyperglycemic symptoms acted both as a barrier and a facilitator in the measurement of glucose levels. Some patients reported that they were no longer aware of the symptoms. They found that performing SMBG helped them to prevent and detect any extreme states. In this case the lack of awareness acted as a facilitator.

'It is difficult for me to estimate how high my glucose levels are. Some people do this perfectly, but I definitely cannot. So, I can guess, and just do whatever. But if my estimate is far off, and the level is either much higher or much lower, then it is no good to me. So, I keep measuring five times per day.'

Other respondents reported that they did not have hypo- or hyperglycemic symptoms. They did not feel the need to test because they felt good. These respondents could have no hypoglycemia or hyperglycemia. But it could also be a lack of awareness. Awareness of the symptoms showed a similar duality. There were respondents who used SMBG as a check to confirm their symptoms, where the awareness acted as a facilitator. Other respondents did not need confirmation, because they already felt the symptoms.

'When things are fine in the morning, and I feel good that day, I don't worry about testing. I go with how I'm feeling. And that works for me.'

##### Knowledge, including misconceptions

According to the respondents, their knowledge regarding self-monitoring had increased as a result of their own experience, education, reading the provided literature, and listening to the anecdotal experiences of other patients. Some respondents reported that the initial phase was of particular importance as it formed the foundation for their goals and perceptions.

'I was told: you can live a normal life with diabetes. That's why I wasn't focused on properly regulating my blood sugar and doing my own monitoring. For me I felt it was a license not to be focused on my diabetes. This effect may persist for a long time.'

It became clear from the interviews that experiential knowledge played an important role in the use of SMBG. Most of the respondents had been using SMBG for a number of years. They felt that they were unique in the way that they reacted to it. They had learned from their experiences and knew what was realistic for them. They did not feel that their results were comparable to others. This could lead to frustration.

'But the stories you hear such as be careful with exercising because your blood sugar can go down as much as one point. Then you'll see that with me, it actually goes up. Everything is different than what I hear. That is also what frustrates me so much. I can't explain it.'

For other respondents this uniqueness led to resignation to their current situation. Better glycemic control was not possible for them.

'The doctor told me: "all right, your glucose concentrations are now between 10 and 15 mmol/l. Just try to get the readings between 6 and 10 or 11. That would be quite an improvement." But that is quite a step forward. Some days everything is going all right and I have good glucose concentrations. But I just accept that this is not always the case. I just know that for me it is difficult to achieve good glycemic control. I do not try to get better readings. It will never be perfect and it is better to accept that.'

There were some respondents who mentioned that they had had misconceptions. For example, what constituted a good value? In almost every public advertisement for a blood glucose meter, a value of approximately 5.8 mmol/l was depicted on the meter. Although other target values had been discussed with the health care provider, several of the respondents reported that they nonetheless wanted to achieve these published values which led to a high frequency of glucose monitoring. They then felt frustrated when they were not able to achieve these results. Others were able adjust their expectations once they had consulted with their health care provider.

##### Life-events

Major life-events, such as having a baby, can cause patients to be more concerned about their glycemic control, which in turn leads to a higher frequency of glucose monitoring. Other events, such as a serious illness in the family, can act as distractors from the diabetes, leading to a decreased frequency of monitoring.

#### Contextual factors

##### Social support

The social support desired by many of the respondents was described as 'being concerned without intervening'. Diabetes is part of the respondents' personal lives. They have to deal with it themselves. Because they do not want to burden the people in their social environment, they use SMBG to keep the diabetes under control. They would like support in the form of recognition of the impact of having diabetes and having to perform SMBG. Furthermore, although contact with other patients with diabetes was sometimes found helpful, there were also respondents who did not relate to the perceptions and behaviours experienced by other patients.

##### Contact with healthcare provider: discrepancies in perspectives

'They really want you to do everything to achieve good glycemic control. The stricter the better. But then I think: That all sounds pretty good on paper, but for me it's like well, ok, you feel better with somewhat higher sugar levels? Yes, actually I do. Well, then I'll do it this way.'

Many respondents reported feeling a certain tension between their goals and perceptions of SMBG and that of their health care providers. According to the respondents, professionals focused more on strict glycemic control, whereas the respondents had to balance glycemic control with their quality of life. Respondents mentioned that professionals could support them in their self-monitoring practices by paying attention to the patients' goals, perceptions, and their readings (not only to HbA1c levels). If the health care professionals were to recognize the uniqueness of the individual patient and tailor the care and the design of glucose regimens to them, the patient would feel better supported.

Some respondents reported feeling ashamed that they did not meet the goals set for them by their health care provider, and that they were obtaining 'poor readings', despite the effort they were putting into it. Sometimes they did not report their results, because they were afraid of the negative response they would receive from the health care provider. Others did not discuss their measurements and did not bring up questions and concerns, as they were afraid that they would have to change their lifestyle as a result. Other respondents claimed to be nonchalant, and accepted that their measurements would not be discussed.

'I'm bad at keeping track. I measure, but I don't keep track. So when I visit the doctor, I come without a record. The doctor only looks at the average values and those are good. Whereas I know, and I think I've mentioned it to the doctor, that it's because of the peaks and valleys that the average ends up good. But nothing was done with this information. Nothing changed until the moment that I, myself, started saying, come on guys, something has to be done. Then things started happening, and they started thinking along with me. Before now, I left things as they were as well. I was pretty nonchalant. I figured, if the average value was good, and I had the feeling that the morning began well, and the evening ended well, everything was okay. If everything was approximately correct, no harm was being done.'

The respondents recognized that they were responsible for their health and for taking care of their diabetes including self-monitoring. Health care providers could, however, provide support, but when the respondents were not satisfied with the delivered care, they seldom discussed it with the health care provider, because they wanted to preserve a positive atmosphere.

### Differences between patients with type 1 and type 2 diabetes

There were a number of similarities between the respondents with type 1 and type 2 diabetes, but the emphasis on separate factors was sometimes different.

Respondents with type 2 diabetes were less focused on adjusting the insulin dose. They were more focused on adjusting food intake and their lifestyle, including exercise. Usually, they only changed their insulin dose in consultation with the health care provider. They were more sensitive to the advice of their health care providers, and they were more attentive in general. Furthermore, their glucose monitoring showed more variation in both timing and frequency. They were more likely to report that they wanted to enjoy their lives without having to continually focus on their diabetes. Respondents with type 1 diabetes performed SMBG with greater regularity and an increased frequency when compared to the patients with type 2 diabetes. The diabetes was also more of an integral part of their lives.

Respondents with type 2 diabetes experienced more complications associated with their diabetes, although this did not seem to have a great effect on the frequency with which they monitored their blood glucose. They also experienced more frustration, not understanding unexpected readings when they monitored their blood glucose. Respondents with type 1 diabetes seemed to be more resigned to their glycemic control. Finally, respondents with type 1 diabetes reported more often that they were no longer aware of hypo- and hyperglycemic symptoms. As result, they felt more dependent on SMBG for feedback about the status of their diabetes.

## Discussion

### SMBG from the patients' perspective

This study investigated the patients' perspective of SMBG. The outcomes of our study show that there is a wide variety in the performance of SMBG, as a result of a complexity of factors. These factors include the patients' perception, his/her goals, and personal and contextual factors. Patients did not always perceive SMBG as a positive tool, which would enable them to achieve good glycemic control. Patients mentioned many experiences which support a negative perception of SMBG, which made it more difficult to perform SMBG. They also felt that health care providers were predominantly focused on good glycemic control. The patients experienced the tension between achieving good glycemic control and quality of life. As a result, the patients tailored the performance of SMBG to their perceptions and personal goals. Personal and contextual factors were identified as being either barriers or facilitators to SMBG depending on the patients' perspective. Most of these factors proved to be dynamic, as they were apt to change over time, sometimes as the result of an intervention by the health care provider. We developed an SMBG model which gives a representation of all the identified influencing factors and their interrelations.

The SMBG model is a specialized version of the General Self-management Model, which gives a representation of the factors, which must be considered when discussing self-management [25]. The SMBG model gives additional information and more practical content to the factors. Furthermore, the factors are arranged in a different way. For example, in the general model, the interaction between the patient and the health care provider plays a central role. In the SMBG model, this relationship is only one of the contextual factors which influences the patients' perspective.

Factors identified in our study such as pain, frustration, depression, knowledge level, treatment complexity, social support, and contact with the health care provider have also been reported in previous studies, including the recent study of Fisher et al. [8,10,12-21,27,28]. Fisher has investigated the knowledge and behavioural skills in more detail [15]. He concluded that more research is necessary to understand the factors, especially among patients with type 2 diabetes. Our study does offer a broad perspective and more insight into the influencing factors and the relationship between them among patients with type 1 and insulin-treated patients with type 2 diabetes. For example, we show that whereas patients with depression tend to tailor their self-monitoring goals to their life situation, the frequency of SMBG did not always change. In our study, the patients' perception of SMBG is described as a separate factor, and is presented as occurring on a continuum from positive to negative. We investigated the deliberate choices made by patients regarding the goals and performances of SMBG, and looked at the logic which governed their decisions.

In other studies, factors have been reported as being either barriers or facilitators to the performance of SMBG. Our study revealed that there are additional nuances which must be considered. Personal and contextual factors had either a positive or a negative effect, depending on the patients' perspective.

### SMBG: small effort, great pleasure?

SMBG is not only positively presented in professional guidelines [6,7,29,30]. Manufacturers also present SMBG as a tool with which diabetes may be controlled, thereby allowing patients to live a normal life. In their advertisements, the readings on the glucose meters usually give values of approximately 5.8 mmol/l while people in the commercial are smiling, looking relaxed and are enjoying life. The meters appear user friendly, the results are almost instantaneous, and apparently almost anybody can use them. Our findings agree with those reported in other studies, including one study in children with diabetes, that SMBG in daily practice is not just a small effort, and it does not always guarantee the pleasure seen in the commercials [13,31]. From the patients' perspective, SMBG is more complex, and the diabetes is often considered difficult to manage. These overly positive representations do not do justice to the visible and invisible work that patients with diabetes have to do nor do they acknowledge the unexpected and sometimes negative readings with which the patients are sometimes confronted despite their efforts. Patients with diabetes need recognition for these efforts and recognition for the impact that SMBG has on their daily lives.

### Contact with the health care provider: duel or duet?

Increasingly, in health care, persons with a chronic disease are given a more central role in their treatment. The patient is responsible for the way in which he/she deals with the disease process. He/she is co-director of his/her own care plan, and is expected to be actively involved. This includes being able to discuss his/her own interests and needs and organizing his/her own care. With an individual care plan, mutually agreed goals and a treatment plan should be outlined with shared responsibilities. This approach should result in improved health care outcomes and lower costs [8,29-34].

However, in our study, as well as in Dedding's study, a discrepancy in perspectives was found between the patients and the health care providers [31]. According to the respondents, health care providers sometimes set unachievable goals, and persisted in trying to motivate them to attain these goals. While many patients accepted that better glucose concentrations were not feasible for them. Furthermore, the advice did not always concur with the patients' needs. As a result, patients ended up making their own choices regarding blood glucose monitoring. Patients tend not to discuss the poor support or the dissatisfying aspects of their treatment as they are afraid that it would negatively influence either the atmosphere or the treatment itself. Health care providers need to be aware of this so that they can take into account the patients' need to preserve the provider-patient relationship [31,35].

### Differences between patients with type 1 and type 2 diabetes

Vincze et al. reported that patients with type 1 diabetes are more adherent to SMBG than patients with type 2 diabetes. Patients with type 2 diabetes may be less convinced that regularly testing blood glucose levels would lead to positive outcomes [10]. Our study shows that patients with type 1 diabetes were not specifically more adherent to blood glucose monitoring, but they did seem more resigned to their situation, and were more likely to view SMBG as an integral part of their lives. Additionally, more factors were identified which influenced the patients' perspectives of SMBG. For example, patients with type 2 diabetes experienced more frustration when they obtained unexpected results.

### Implications for daily practice

In daily practice, health care providers also have to find a balance between achieving good glycemic control and quality of life. Glycemic control is recommended in the guidelines, it helps prevent long term complications, and is an important indicator for the quality of health care. The patient's quality of life is equally as important. The challenge for the health care provider is to set, in discussion with the patient, glycemic goals which are as strict as possible while maximizing the patient's quality of life. It is not a matter of only listening to the patient's perspective, including his/her goals and needs, but it is a starting point.

The SMBG model can help professionals and patients to understand and discuss each other's perspectives in order to be able to set mutually agreed upon, realistic, and achievable goals. Furthermore, it is important that professionals discuss both the positive and the potentially negative aspects of SMBG from the very start including how to deal with SMBG on a daily basis. This will serve to enhance the patient's feelings of personal control and help to cope with the inevitable disappointments, thereby enhancing positive perceptions of SMBG [16,21,36-38]. The performances can also be individually tailored to the patient. This will allow an optimization of SMBG use and will prevent both unnecessary measurements and costs. After all, there is no evidence supporting a specific frequency of SMBG, which has led to a variety of recommendations in the literature [6,29,30]. SMBG is not a goal in and of itself, but a tool which should be used to achieve optimal glycemic control in an effort to prevent long term complications [21]. Education and training will likely be necessary before the health care providers can optimize their use of this approach and attitude [8].

Regular follow up and evaluation is important as most of the factors affecting the SMBG model are dynamic.

Finally, a new study is started to develop and validate a clinical measurement tool of patients' perspective of SMBG.

### Strengths and limitations of the study

Our patient population consisted of native Dutch patients, therefore the results can not be extrapolated to patients of different ethnicities. Although we followed the principles of purposeful and theoretical sampling, the study participants were asked to participate by their own health care provider which may have led to a selection bias. On the other hand, we were able to select a widely diverse sample. The maximum variation of relevant characteristics made it possible to make full use of comparative analysis theory. Our study not only confirms what has been reported in previous studies, but provides a deeper insight into the perspectives of insulin treated patients with diabetes.

## Conclusions

According to the patients, professionals are positive about the use of SMBG as a tool in order to achieve strict glycemic control. Patients can also, however, perceive SMBG in a negative light. They have to find a balance between achieving glycemic control and maintaining acceptable quality of life. As a result, the patients' use of SMBG is not always in concordance with the recommendations from the health care provider. Patients with type 1 diabetes appeared to be more resigned to their situation and monitoring their blood glucose was a more integral part of their lives than it was for patients with type 2 diabetes. Gaining insight and discussing all the factors involved with SMBG, including personal barriers and facilitators, will help health care providers and patients mutually agree on realistic and achievable goals while tailoring the performance of SMBG on an individual basis. This should result in a more optimal use of SMBG, an improved quality of life, and improved clinical parameters.

## Competing interests

The authors declare that they have no competing interests.

## Authors' contributions

Study design: JH, JvdB; data collection JH; analysis and interpretation of the data JH, MCK, WSW, JvdB; manuscript preparation JH, MCK, WSW, NK, HJGB, JvdB. All authors read and approved the final manuscript.

## Pre-publication history

The pre-publication history for this paper can be accessed here:

http://www.biomedcentral.com/1471-2458/12/167/prepub

## References

[B1] Diabetes Control and Complication Trial Research Group The effect of intensive treatment of diabetes on the development and progression of long-term complications in insulin-dependent diabetes mellitus N Engl J Med 1993 329 977 986 8366922 10.1056/NEJM199309303291401

[B2] Holman RR Paul SK Bethel MA Matthews DR Neil HA 10-year follow-up of intensive glucose control in type 2 diabetes N Engl J Med 2008 359 15 1577 1589 10.1056/NEJMoa0806470 18784090

[B3] Goldstein DE Litle RR Lorenz RA Malone JI Nathan D Peterson CM Sacks DB Tests of glycemia in diabetes Diabetes Care 2004 27 1761 1773 10.2337/diacare.27.7.1761 15220264

[B4] Nathan DM McKitrick C Larkin M Schaffran R Singer DE Glycemic control in diabetes mellitus: have changes in therapy made a difference? Am J Med 1996 100 157 163 10.1016/S0002-9343(97)89453-3 8629649

[B5] Karter AJ Ackerson LM Darbinian JA D'Agostino RB Jr Ferrara A Liu J Selby JV Self-monitoring of blood glucose levels and glycemic control: the Northern California Kaiser Permanente Diabetes Registry Am J Med 2001 111 1 9 11448654 10.1016/s0002-9343(01)00742-2

[B6] Dutch Diabetes Federation Recommendation self monitoring of blood glucose 2003 Amersfoort, NDF http://diabetesfederatie.nl Accessed 1 May 2011

[B7] Dutch Association of Diabetic care professionals Recommendation of self monitoring of blood glucose 2004 http://eadv.nl Accessed 1 May 2011

[B8] World Health Organization Adherence to long-term therapies. Evidence for action 2003 Geneva: WHO

[B9] Bergenstal RM Gavin JR Global Consensus Conference on Glucose Monitoring Panel The role of self-monitoring of blood glucose in the care of patients with diabetes: report of a global consensus conference Am J Med 2005 118 Suppl 9ª 1S 6S 16224936 10.1016/j.amjmed.2005.07.055

[B10] Vincze G Barner JC Lopez D Factors associated with adherence to self-monitoring of blood glucose among persons with diabetes Diabetes Educ 2004 30 1 112 125 10.1177/014572170403000119 14999899

[B11] Davidson J Strategies for improving glycemic control: effective use of glucose monitoring Am J Med 2005 118 Suppl 9A 27S 32S 16224940 10.1016/j.amjmed.2005.07.054

[B12] Mollema ED Snoek FJ Pouwer F Heine RJ van der Ploeg HM Diabetes fear of injecting and self-testing questionnaire. A psychometric evaluation Diabetes Care 2000 23 6 765 769 10.2337/diacare.23.6.765 10840993

[B13] Mol A What diagnostic devices do: the case of blood sugar measurement Theor Med Bioeth 2000 21 1 9 22 10.1023/A:1009999119586 10927966

[B14] Fisher WA Barriers and behaviours in blood glucose monitoring US Endocrine Disease 2007 2 51 53

[B15] Fisher WA Kohut T Schachner H Stenger P Understanding Self-monitoring of blood glucose among individuals with type 1 and type 2 diabetes. An information-motivation-behavioral skills analysis Diabetes Educ 2011 37 85 94 10.1177/0145721710391479 21292622

[B16] Snoek FJ Breaking the barriers to optimal glycaemic control: what physicians need to know from patients' perspectives Int J Clin Prac Suppl 2002 129 80 84 12166612

[B17] Peel E Parry O Douglas M Lawton J Blood glucose self-monitoring in non-insulin-treated type 2 diabetes: a qualitative study of patients' perspectives Br J Gen Pract 2004 54 500 183 188 15006123 PMC1314828

[B18] Peel E Douglas M Lawton J Self-monitoring of blood glucose in type 2 diabetes: longitudinal qualitative study of patients' perspectives BMJ 2007 335 7618 493 10.1136/bmj.39302.444572.DE 17761996 PMC1971180

[B19] Farmer AJ Wade AN French DP Blood Glucose monitoring in type 2 diabetes: a randomised controlled trial Health Technol Assess 2009 13 15 29 40 qualitative interviews 19254484 10.3310/hta13150

[B20] Collins MM Bradley C O'Sullivan T Perry IJ Self-care coping strategies in patients with diabetes: a qualitative exploratory study BMC Endocrin Disord 2009 9 6 10.1186/1472-6823-9-6 PMC2664817 19232113

[B21] Snoek FJ Malanda UL de Wit M Self-monitoring of blood glucose: psychological barriers and benefits Eur Diabetes Nursing 2008 5 3 112 115 10.1002/edn.122

[B22] Holloway CI Wheeler S Qualitative Research in Nursing 2002 2 Oxford: Blackwell Science Ltd

[B23] Cresswell JW Qualitative inquiry and research design. Choosing among five approaches 2007 2 Sage publications, Inc.

[B24] Boeije HR Analysis in qualitative research 2010 Sage publications, Inc.

[B25] The Federation of Patients and Consumer Organisations and the Dutch Institute for Healthcare Improvement The general model of self-management 2011 http://www.zelfmanagement.com/downloads/209/general-model-english.pdf Accessed 31 May

[B26] Wagner EH Austin B Korff von M Schaefer J Reid R Colema K Improving Chronic Illness Care. The Chronic Care Model 2011 http://www.improvingchroniccare.org Accessed 31 May

[B27] Gonzalez JS Peyrot M McCarl LA Collins EM Serpa L Mimiaga MJ Safren SA Depression and diabetes treatment nonadherence: a meta-analysis Diabetes Care 2008 31 12 2398 2403 10.2337/dc08-1341 19033420 PMC2584202

[B28] McGrady ME Laffel L Drotar D Repaske D Hood KK Depressive symptoms and glycemic control in adolescent with type 1 diabetes Diabetes Care 2009 32 5 804 806 10.2337/dc08-2111 19228870 PMC2671131

[B29] Care recommendations: Selfmonitoring of blood glucose- Diabetes UK 2008 2011 http://www.diabetes.org.uk/About_us/Our_Views/Care_recommendations/Self-monitoring_of_blood_glucose Accessed 1 May

[B30] American Diabetes Association Standards of medical care in diabetes, 2009 Diabetes Care 2009 32 Suppl 1 S13 S61 19118286 10.2337/dc09-S013 PMC2613589

[B31] Dedding C The verbal expression of children is often expressed in silence. Participation of children in diabetes care 2010 (Dutch) Bohn Stafleu Van Loghum

[B32] Dutch Diabetes Federation Diabetes Care Standard Amersfoort juli 2007 http://www.diabetesfederatie.nl Accessed 1 May 2011

[B33] Wagner EH Austin BT Davis C Hindmarsh M Schaefer J Bonomi A Improving chronic illness care: translating evidence into action Heal Aff 2001 20 6 64 78 10.1377/hlthaff.20.6.64 11816692

[B34] von Korff M Grumann J Schaefer J Curry SJ Wagner EH Collaborative management of chronic illness Ann Intern Med 1997 127 12 1097 1102 9412313 10.7326/0003-4819-127-12-199712150-00008

[B35] Ciechanowski P Katon WJ The interpersonal experience of health care through the eyes of patients with diabetes Social Science Medicine 2006 63 3067 3079 10.1016/j.socscimed.2006.08.002 16997440

[B36] Dunn SM Psychological issues in diabetes management: (I) Blood glucose monitoring and learned helplessness Practical Diabetes 1987 4 108 110 10.1002/pdi.1960040304

[B37] de Ridder DTD de Wit JBF Self-regulation in health behaviour, chapter 2 and 7 2006 Chichester: Wiley

[B38] Thoolen BJ Ridder de D Bensing J Gorter K Rutten G Beyond good intentions: the role of proactive coping in achieving sustained behavioral change in the context of diabetes management Psychol Health 2009 24 237 254 10.1080/08870440701864504 20204991

